# Development of Prototype Low-Cost QTSS™ Wearable Flexible More Enviro-Friendly Pressure, Shear, and Friction Sensors for Dynamic Prosthetic Fit Monitoring

**DOI:** 10.3390/s21113764

**Published:** 2021-05-28

**Authors:** Valter Dejke, Mattias P. Eng, Klas Brinkfeldt, Josephine Charnley, David Lussey, Chris Lussey

**Affiliations:** 1 RISE Research Institutes of Sweden, Argongatan 30, 431 53 Mölndal, Sweden; valter.dejke@ri.se (V.D.); mattias.eng@ri.se (M.P.E.); klas.brinkfeldt@ri.se (K.B.); 2 Lusstech, NETpark, Thomas Wright Way, Sedgefield TS21 3FD, UK; david.lussey@lusstech.com (D.L.); chris.lussey@lusstech.com (C.L.)

**Keywords:** Quantum Technology Supersensors™, QTSS™, quantum materials, quantum tunnelling conduction, composite materials, pressure sensor, shear sensor, loss of friction sensor, flexible sensor, wearable sensor

## Abstract

There is a current healthcare need for improved prosthetic socket fit provision for the masses using low-cost and simple to manufacture sensors that can measure pressure, shear, and friction. There is also a need to address society’s increasing concerns regarding the environmental impact of electronics and IoT devices. Prototype thin, low-cost, and low-weight pressure, shear, and loss of friction sensors have been developed and assembled for trans-femoral amputees. These flexible and conformable sensors are simple to manufacture and utilize more enviro-friendly novel magnetite-based QTSS™ (Quantum Technology Supersensor™) quantum materials. They have undergone some initial tests on flat and curved surfaces in a pilot amputee trial, which are presented in this paper. These initial findings indicate that the prototype pressure sensor strip is capable of measuring pressure both on flat and curved socket surfaces in a pilot amputee trial. They have also demonstrated that the prototype shear sensor can indicate increasing shear forces, the resultant direction of the shear forces, and loss of friction/slippage events. Further testing, amputee trials, and ongoing optimization is continuing as part of the SocketSense project to assist prosthetic comfort and fit.

## 1. Introduction

Limb amputations cause serious physical disabilities worldwide and compromise peoples’ quality of life globally. In the USA alone, in 2005, there were an estimated 1.6 million persons living with limb loss in the United States, with 19% to 21% of those individuals living with a transfemoral (above knee) lower limb amputation [1]. These numbers could double by 2050 due to the increasing rates of vascular disease and diabetes [2,3].

Limb prostheses can reduce the negative impact of such disabilities and attempt to restore normal functionality and amputee autonomy as much as possible. Serious attention has to be paid though to the prosthetic socket design in order to achieve a functional and comfortable prosthesis. The socket for lower-limb amputees is a critical human–machine interface between an amputee’s stump and the prosthetic limb device. A good socket must achieve satisfactory load transmission, stability, and efficient control for mobility. In addition, the biomechanical understanding of the interaction between the prosthetic socket and the residual limb is fundamental and key to the success or failure of the prosthetic limb itself [4]. In lower limb amputees, the so-called “pistoning effect” occurs, which is a vertical relative movement of the stump inside the socket that can sometimes be abrasive. Optimizing the interaction between the stump and the inner surface of the socket is essential for the long-term acceptability, comfort, and good functionality of the prosthesis [5].

A survey by the Amputee Coalition on random amputees found that 35.3% of amputees with a prosthesis did not wear it regularly due to comfort issues [6]. Despite advances in prosthetics, many amputees still reject their prosthesis or show a low satisfaction level. This is mainly as a consequence of socket-related issues, such as poor comfort, reduced biomechanical functionality, and hampered control [5,7]. In addition, skin lesions also occur in 63–82% of lower limb amputees, causing a prostheses abandon rate estimated at around 25–57% [5,8,9].

Poor socket fit and misalignment can result in extrinsic stump pain. Large pressure gradients within the socket can cause soft tissue inflammation, and bursitis may occur at weight-bearing regions. In many cases, reshaping of the prosthetic socket to reduce the pressure gradients can resolve these complications. Due to the nature of prosthetic sockets, skin ventilation is hindered, and perspiration is trapped, which can lead to friction, skin maceration, tissue destruction, and ulceration. Continued rubbing and pressure is capable of producing tissue destruction. Stump skin often becomes macerated or worn away by poor prosthetic fit or alignment. The importance of early recognition and treatment of skin lesions on the residual limbs of amputees cannot be over emphasized [10].

Therefore, there is a continuing healthcare need for an improved prosthetic socket provision technique using sensing technology and real-time monitoring at the stump–socket interface [5]. To date, the most utilized commercial sensing systems for performing pressure measurements in the prosthetics field in clinical settings are the piezo-resistive F-socket System (Tekscan Inc., Boston, MA, USA) and the capacitive Novel System (Novel Electronics Inc., Saint Paul, MN, USA). Piezo-resistive sensors which are based on percolative conduction mechanisms can be designed to be thin and flexible, but they have a smaller dynamic range than those based on quantum conduction mechanisms. They are also dependent on ‘area change’, which can cause problems when attempting to measure on curved surfaces as opposed to flat surfaces. Capacitive sensors can also be thin and flexible, but they require complex electronics and can suffer from cross-talk noise within matrix configurations [5]. The main issue though for both these two commercial systems is that they are very expensive. Therefore, they are not used by the masses despite the fact that there is an identified healthcare need for improved prosthetic fit. Neither do these products address both friction and shear, which is a major cause of tissue damage and skin lesions for amputees, so being able to identify this early to prevent tissue damage would be extremely useful, save costs, and reduce the prosthesis abandonment rate [5].

Current lower limb socket provision is based on a skilled and experienced prosthetist determining the socket design based on a subjective human ‘touch and feel’ technique. Useful assistance could be provided to prosthetists via retrofittable sensors being placed in the socket. It is recognized that novel printable polymer sensors with flexibility could provide such a sensing solution [11].

Thin, light-weight, flexible, wearable prototype QTSS™ pressure, shear, and loss of friction/slippage sensors have been developed to be retrofitted into a socket for dynamic prosthetic fit monitoring as part of the SocketSense project [12]. They are to improve the comfort and fit of trans-femoral (i.e., above knee) prosthetic sockets. The data acquired at the stump–socket interface can be used as input for biomechanical models showing an output of optimal socket design to aid prosthestists in a more quantitative scientific procedure than is currently utilized.

To fully exploit the potential of flexible wearable sensors and to enable mass use, the manufacturability of sensors also has to be simple and processes more easily scalable for Large Area Electronics (LAE) [13]. The pushing of technology barriers in printed flexible wearable electronics is required to enable easy manufacture worldwide. The QTSS™ sensors have been developed with new smart multi-functional quantum materials [14] to achieve a low-cost, simple, and easily manufacturable solution (so scalable for LAE purposes) to assist with and monitor prosthetic fit. In the future, the QTSS™ pressure and shear sensors could also be combined into one low-cost simple printed strip.

Electronic assemblies currently contain many elements known to cause issues with human health and damage the environment [15]. Over recent years, there has been a massive shift of public awareness regarding the need to improve this and to consider these issues during the production and use of new devices required to address societal needs, particularly in the printed electronics field [16]. The Organic and Printed Electronics Association references the Brundtland Report 1987, where sustainable development is defined as ‘development that meets the needs of the present without compromising the ability of future generations to meet their own needs’ [17]. QTSS™ materials are patented quantum materials based on enviro-friendly natural magnetite developed to enable flexible electronic sensors to be made more environmentally friendly [14]. Magnetite is an abundant and very green material and easy to identify for recovery and recycling in an LAE circular economy [16]. Quantum materials also have the added benefit of energy efficiency during use [18].

This article discusses the initial development, assembly, and testing of prototype low-cost QTSS™ wearable, flexible, and more enviro-friendly pressure, shear, and friction sensors for dynamic prosthetic fit monitoring. It describes the QTSS™ quantum materials used and how the prototype sensors were assembled. It goes on to discuss the experimental setups for the initial testing of the pressure sensors on the flat and on socket curvatures and the shear and loss of friction sensors in a test rig. Initial results are presented for the pressure sensors dynamic single loading repeatability (pressure versus resistance), the influence of fast dynamic load cycling, the effect of socket curvatures, and for the shear and loss of friction sensor. Finally, some results of an initial pilot amputee trial are presented. Further testing and optimization is continuing as part of the SocketSense project [12]. Further work will be carried out on the analysis and comparisons of other sensors and technologies which are outside the scope of this article.

## 2. Materials—QTSS™ Quantum Materials

QTSS™ screen printable inks are produced by Quantum Technology Supersensors and harness ‘nature’s’ sub-atomic quantum conduction mechanisms based on quantum mechanics principles rather than classical mechanics principles [14]. They are patented novel compositions of matter.

**Figure 1:**
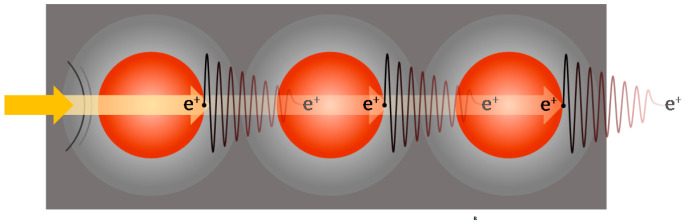
Tunneling of electrons through insulative barriers around the magnetite particles.

Conduction in these sensing materials is achieved via tunneling of electrons through insulative barriers around the magnetite particles (see Figure 1 below). Quantum conduction allows these quantum materials to sense across the whole range of electrical conductivity, from insulator to conductor, and it provides other unique and very useful electrical effects [18].

**Figure 2:**
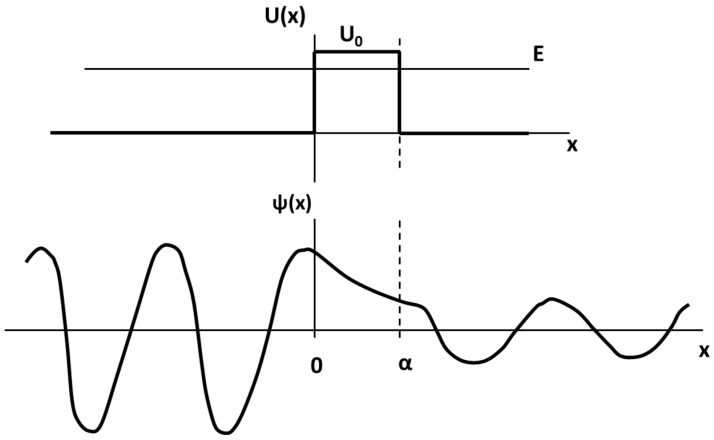
Tunneling—wave function behavior.

Tunneling is a consequence of the wave-particle duality of the electron. When a ‘free’ electron (e.g., an electron in the conduction band of a metal moving under the influence of an external electric field) impinges on a non-conducting barrier of width α with a height (U_0_), which is greater than that of the energy of the electron (E), illustrated in the top part of Figure 2, the wave function behaves as follows:

Within the barrier, the wave function decays exponentially as illustrated in the bottom part of Figure 2. The ratio of the wave function amplitude squared on either side of the barrier is called the transmission coefficient (T) and is a measure of the probability that the electron penetrates the barrier. The transmission coefficient is given by Equations (1) and (2):

(1) $$T=e^{-2\kappa \alpha }$$

(2) $$\kappa =\sqrt{2m\left(U_{0}-E\right)/{ћ}^{2}}$$

where m is the electron mass and ħ is Planck’s constant divided by 2π. Thus, this transmission coefficient is also the fraction of all incident electrons of energy E that penetrate the barrier. Thus, in macroscopic terms, it determines the fraction of an incident current transmitted through the insulating barrier. When the barrier is very thin, i.e., of atomic dimensions, T is approximately one, and the barrier does not impede the current.

**Figure 3:**
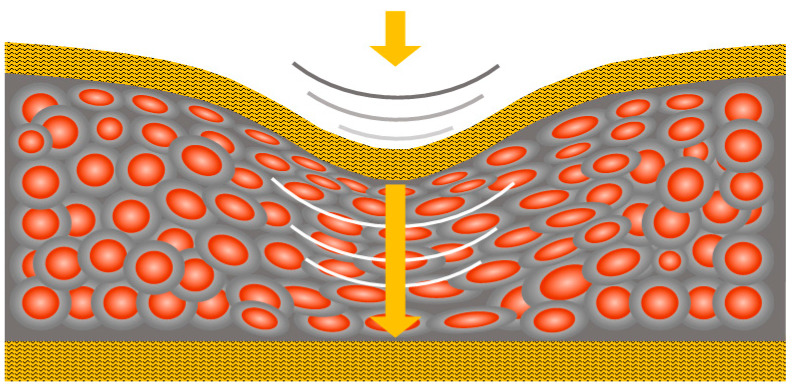
Conduction occurs ‘through’ the materials only at the point of applied pressure or stimu-lus.

It is also due to the quantum conduction mechanism that QTSS™ materials have the ability to conduct ‘through’ the materials only at the point of applied pressure or stimulus (see Figure 3 below) and not throughout the whole body of the material, which is the case for most conductive materials. As these quantum materials are insulators in their normal state (i.e., the quiescent resistance of these materials is extremely high), they are ‘energy efficient’ during use [18].

Quantum conduction allows the range, sensitivity, and control to provide a real-world pressure-sensing experience. As pressure is applied, a massive resistance change occurs over many orders of magnitude (from insulator to metallic-like conductor), and the resistance change is in proportion to the pressure applied. This sensitivity and large dynamic range of QTSS™ materials pressure-sensing capability is different to standard percolative piezo-resistive pressure sensors, which have a smaller dynamic range and are dependent on ‘area change’, so they operate in an entirely different way that is less conducive for curved surfaces. The low latency associated with quantum conduction also enables an ‘instantaneous’ response and the anisotropic nature of QTSS™ materials allows simplified sensor designs [18].

## 3. Methods and Results

### 3.1. Assembly of Prototype QTSS™ Pressure Sensor Strip

**Figure 4:**
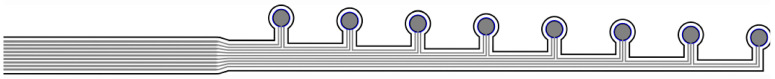
The prototype QTSS™ pressure sensor strip.

The prototype QTSS™ pressure sensor strip was designed to include multiple points of sensing (i.e., 8 individual pressure sensing sensels). It is shown in Figure 4 below. These multi-point sensor strips consist of two screen-printed poly(ethylene terephthalate) (PET) layers adhered together with double-sided adhesive tape. The sensor strip can be trimmed to any size depending on the length of the socket and the placement of the individual sensels. Side 1 is screen-printed with silver ink and QTSS™ pressure sensing ink and Side 2 is simply printed with silver ink. As pressure is applied to a sensel, the QTSS™ pressure sensing ink changes from insulator to conductor in proportion to the pressure applied, with its resistance decreasing. A very straightforward screen-printed design has been utilized, which allows for easy mass production. For this particular application, the final prototype sensor has been designed with a sensitivity to respond to pressures up to around 300 kPa.

**Figure 5:**
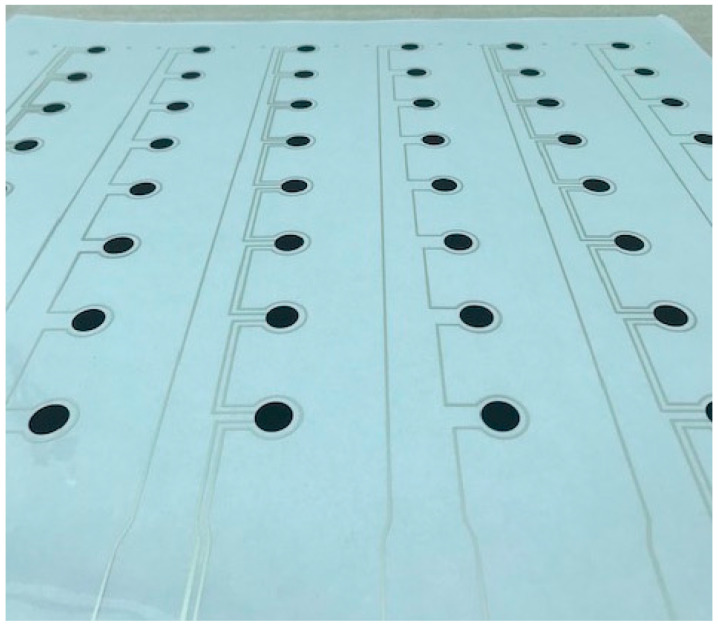
PET sheet screen-printed with multiples of the QTSS™ prototype pressure sensor strip design.

Figure 5 shows an example of a PET sheet screen-printed with multiples of the QTSS™ prototype pressure sensor strip design.

**Figure 6:**
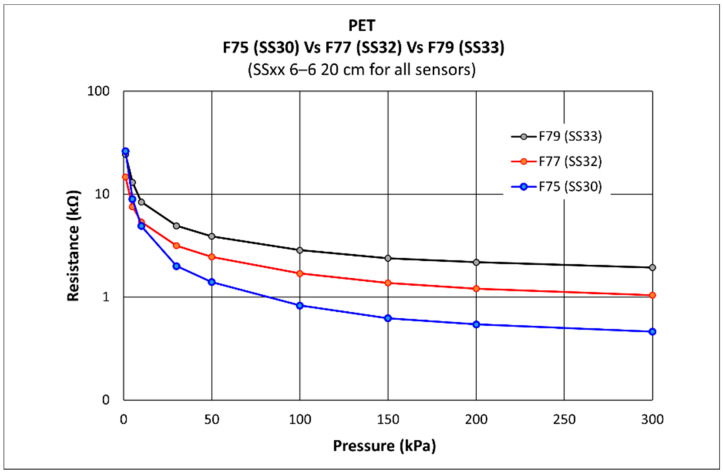
A comparison on ‘PET’ of QTSS™ F75 ink vs. QTSS™ F77 ink vs. QTSS™ F79 ink sensitivities.

The QTSS™ ink has been experimentally chosen for its sensitivity for the pressure range required for this particular application and its use on PET substrate. The graph below (Figure 6) shows a comparison on ‘PET’ of QTSS™ F75 ink vs. QTSS™ F77 ink vs. QTSS™ F79 ink sensitivities. QTSS™ F75 ink was selected for this application.

**Figure 7:**
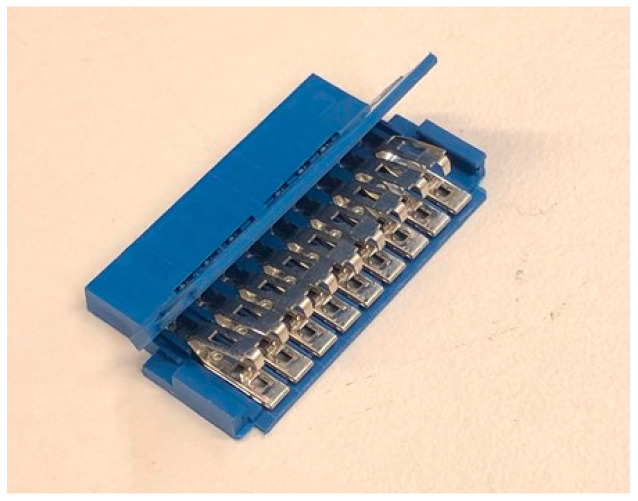
A robust 2.54 mm (nine-way female) Amphenol connector.

A robust 2.54 mm (9-way female) Amphenol FCI IDC connector that could be crimped on the sensor strips to eliminate potential poor connection issues was selected for the QTSS™ pressure sensor strips (shown in Figure 7 below).

### 3.2. Experimental Setup for Testing the QTSS™ Pressure Sensors on a ‘Flat’ Surface

#### 3.2.1. Testing of Pressure vs. Resistance during Single Loadings

**Figure 8:**
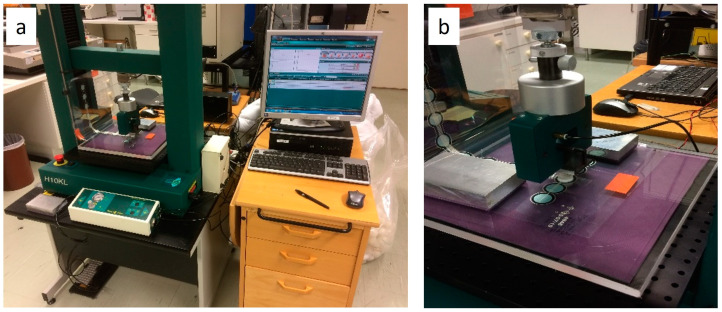
Tinius Olsen tensile tester setup for pressure vs. resistance testing on the flat. Overview of the setup (**a**) and a zoomed in view on the testing of a sensor strip with the contact plate (**b**).

**Figure 9:**
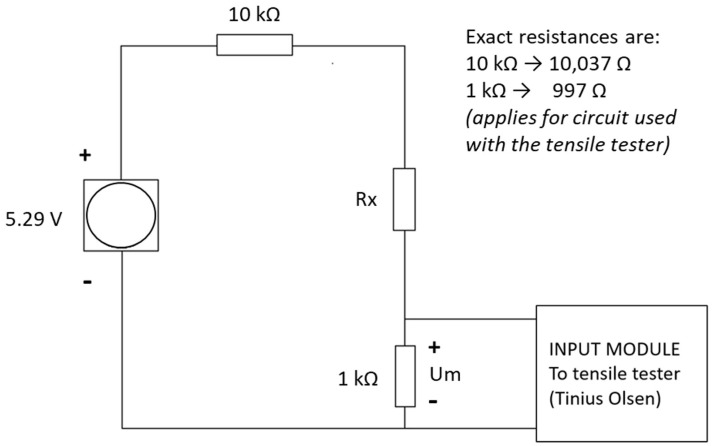
The measuring circuit diagram for dynamic pressure vs. resistance testing on the flat.

Initial dynamic pressure vs. resistance testing was carried out on the QTSS™ pressure sensors up to 300 KPa using a deformation controlled Tinius Olsen tensile tester (loading speed: 20 mm/min) (see Figure 8 below). QTSS™ materials operate at low voltages and currents and therefore, for initial testing, the low-voltage divider circuit shown in Figure 9 below was used with a voltage source of 5.29 V and current of 500 µA max.

A 20 mm diameter circular contact plate made of hard PMMA 4 mm thick plastic with a 5 mm thick white foam (soft Ortoliner from Össur, Iceland) on top was used to replicate a hard socket surface and a soft silicon liner. This test set up was used throughout testing to ensure realistic testing conditions.

**Figure 10:**
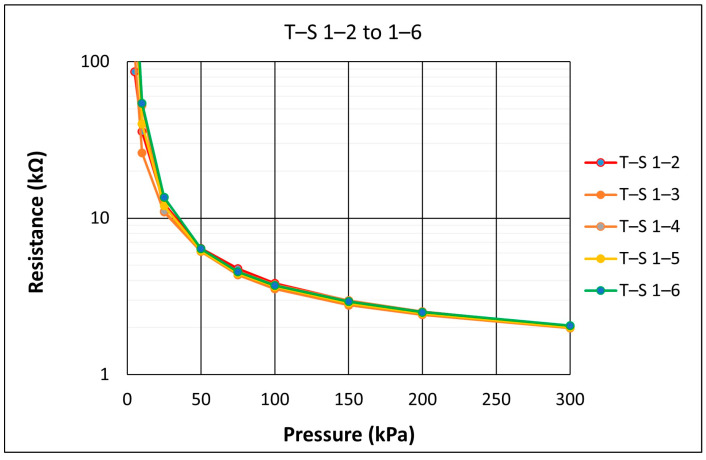
An example of a pressure vs. resistance repeatability test graph carried out on one QTSS™ sensel.

The following pressure versus resistance graph in Figure 10 is an example of a repeatability test carried out on one QTSS™ pressure sensing sensel using single loadings. A pressure of 300 kPa was applied whilst measuring the sensor output, and then, this test was repeated 5 times at a loading speed of 20 mm/min. The first ‘conditioning’ load where the ink stack gets initially compressed was disregarded. At a resistance of 4 kΩ (within the relevant pressure range for this particular application of 75–100 kPa), the standard deviation for this sensel was only 4.4%, so its repeatability accuracy was 95.6% and considered a good repeatability for a low-cost simple printed flexible sensor.

#### 3.2.2. Testing of Pressure vs. Resistance during Dynamic Load Cycling

**Figure 11:**
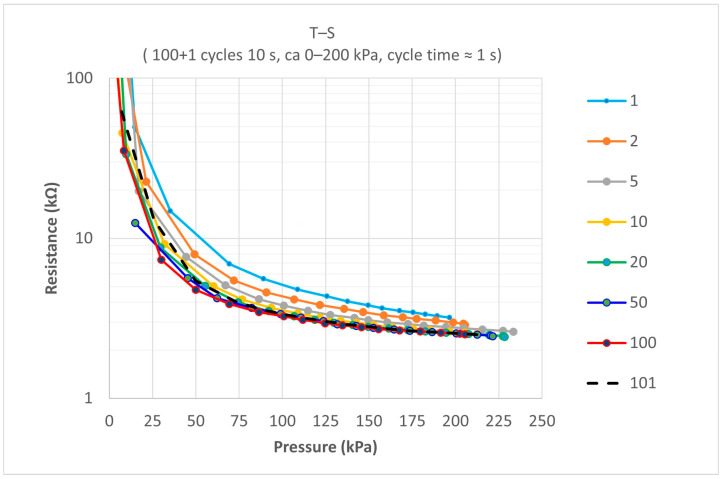
An example of test results for extracted P vs. R curves from within the sets of 101 load cycles carried out on one QTSS™ sensel.

The same QTSS™ sensel was used to carry out testing on the influence of fast dynamic load cycling, because in this particular application, there will be fast dynamic cycling of loads within a socket. A pressure of 0 to 200 kPa was applied in 100 cycles with a frequency of 1 Hz, and a final load cycle was conducted 10 s after the 100th load cycle. The results for extracted pressure versus resistance curves from within the sets of 101 load cycles for this sensel are shown in Figure 11 below. Curves were extracted for the following load cycles: 1, 2, 5, 10, 20, 50, 100, and 101. The first couple of loads were considered ‘conditioning’ loads.

All flexible polymer substrate sensors due to their visco-elastic nature will have some corresponding pressure reading differences for the same resistance values when dynamic load cycling takes place, but it is beneficial for these to be as low as possible. The corresponding pressure reading differences for the same resistance values of the 100th curve versus the 101th curve were found to be only 7 kPa at the 100 kPa resistance value (which is within the relevant pressure range for this particular application) and the prototype QTSS™ sensel showed a good repeatability accuracy during this ‘dynamic cycling’ testing.

### 3.3. Experimental Setup for Testing ‘In Socket’

**Figure 12:**
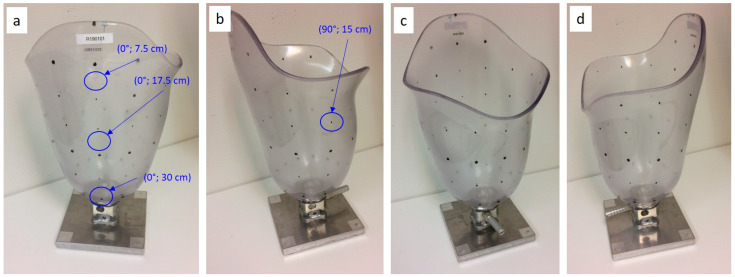
The test socket with coordinates applied to allow individual sensor sensels to be placed in identifiable locations, shown from the lateral (**a**), posterior (**b**), medial (**c**), and anterior view (**d**).

As sockets vary in geometry and contain curved surfaces, to create realistic test conditions, a socket was attached to a base to use for testing, and coordinates were applied to this socket to allow individual sensor sensels to be placed in known and identifiable locations during the in-socket testing, as shown in Figure 12 below.

#### 3.3.1. Resistance Measuring Circuit for ‘In-Socket’ Testing

**Figure 13:**
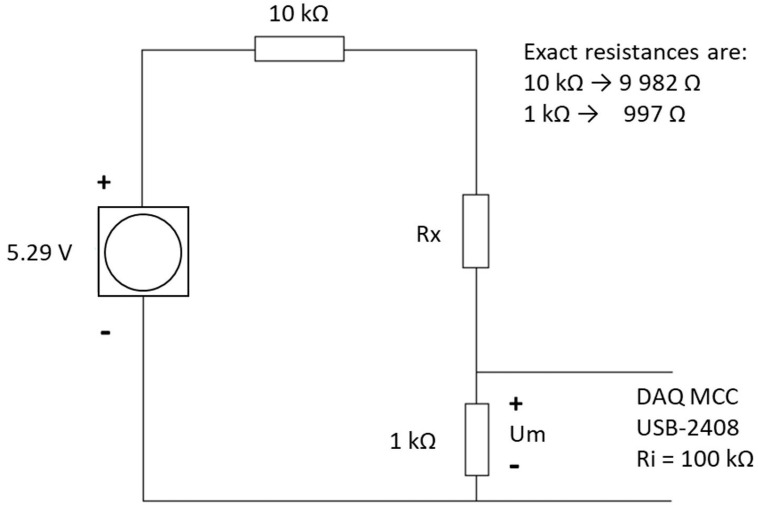
The measuring circuit diagram for the ‘in-socket’ measurements.

The measuring circuit diagram shown in Figure 13 below and the following equipment was used for the ‘in-socket’ testing: A unit containing an A to D convertor; a measurement computing USB-2408; an A to D converter having type number ADS1256; a measuring Lenovo ThinkPad T420s laptop; a transformer generating 5.29 V. The resistors used in the measuring circuit were: (a) R1: 997 Ω (in series with the sensor, over which the voltage is measured) and (b) R2: 9982 Ω (resistor also in series with the sensor).

#### 3.3.2. Simulating the Pressures Caused by a Residual Limb

**Figure 14:**
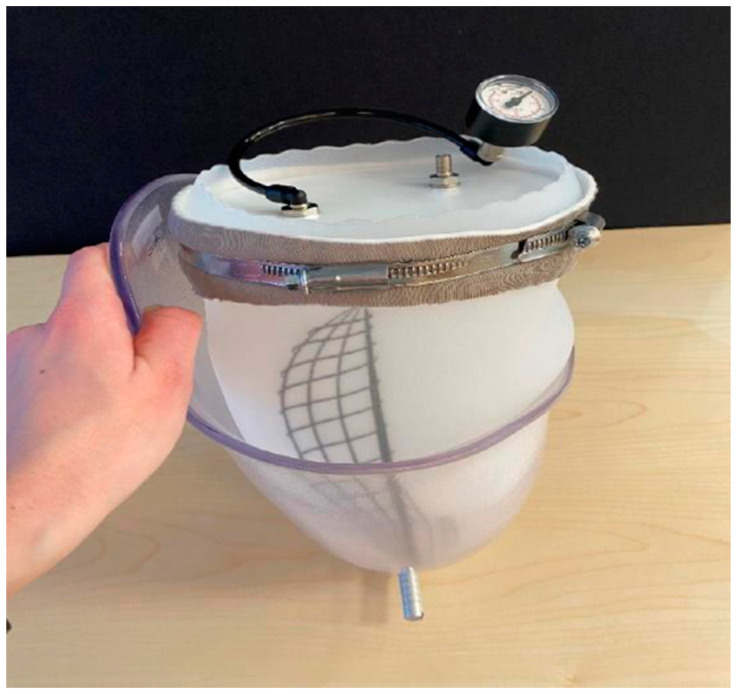
The liner balloon with a top plate.

**Figure 15:**
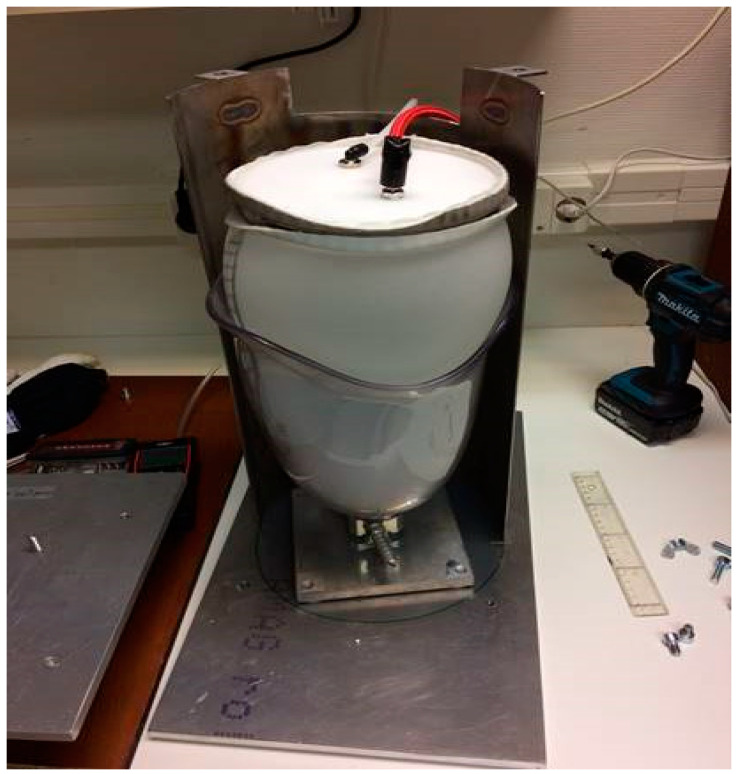
The liner balloon test setup on the base.

**Figure 16:**
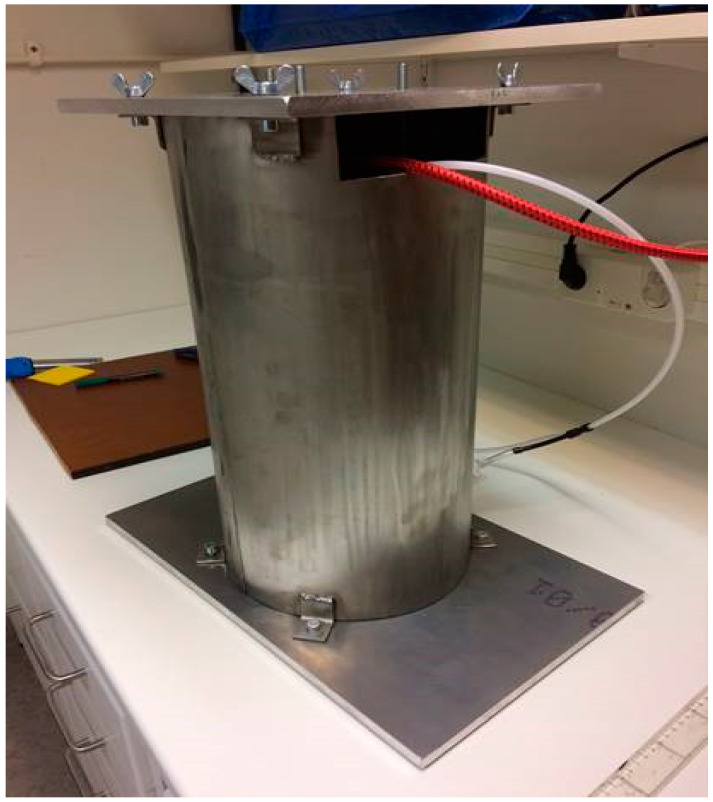
The metal encasement arrangement.

In order to be able to test the sensors capabilities on socket curvatures, a liner balloon was used to simulate the pressure from a residual limb in the socket. This arrangement was not aimed at simulating a real stump (which would be used in the later amputee pilot trial) but would allow known pressures to be applied to the inside of the curved socket walls. This liner balloon was made using an ordinary prosthetic liner with a top plate added, enabling an air-tight volume to be formed. During measurements of pressure sensors in the socket, this balloon was placed inside the socket and pressurized in a controlled manner. In this way, an arbitrary pressure could be applied on the pressure sensors adhered to the inside wall of the socket, which could then be tested under relatively realistic conditions on the curved surfaces. The pressure applied to the sensors was considered equal to the air pressure inside the liner balloon. A metal encasement arrangement was created for safety reasons (in case of liner balloon explosion) and to prevent the liner balloon from expanding uncontrollably above the socket brim, leading to unpredictable pressure conditions in the socket. See Figure 14, Figure 15 and Figure 16 below.

#### 3.3.3. The Test Results for ‘In-Socket’ Testing

**Figure 17:**
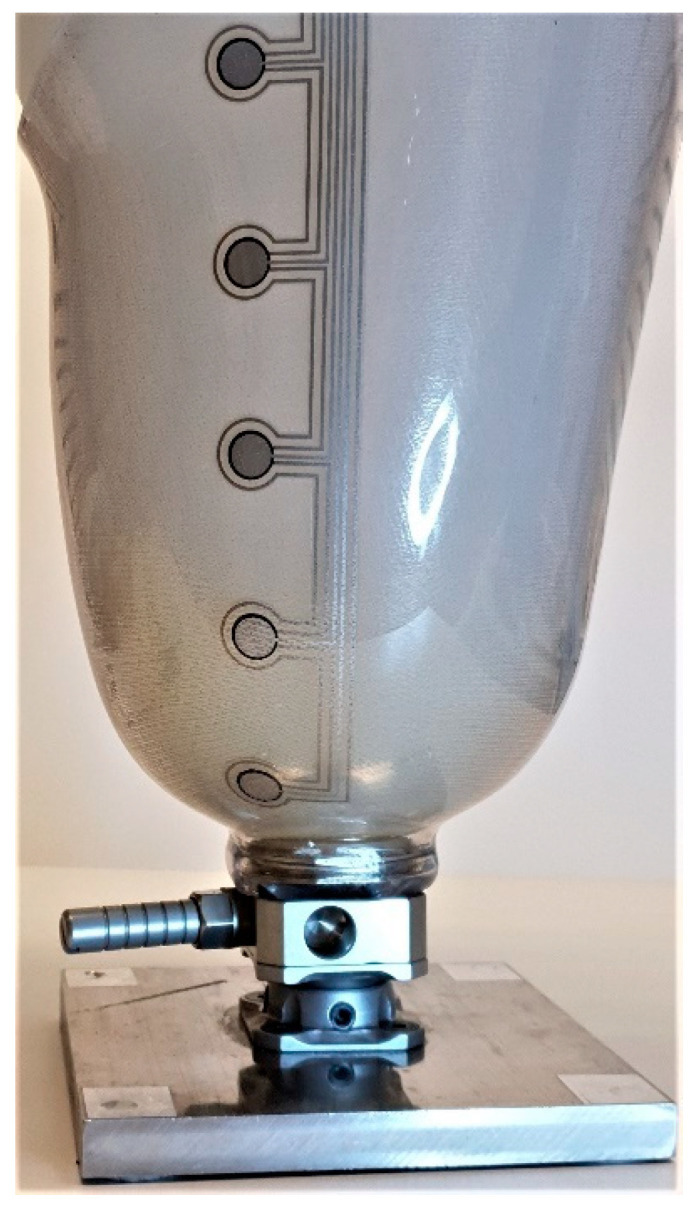
A QTSS™ pressure sensor inside a socket.

A prototype QTSS™ pressure sensor (SS54) was used to carry out the following in-socket tests in order to compare a graphed result for a flat test of the sensor with the ‘in-socket’ tests when the same sensor is placed on a surface with a large and relatively small curvature respectively inside the socket. Figure 17 below shows an example of a flexible and conformable QTSS™ pressure sensor placed inside a socket.

**Figure 18:**
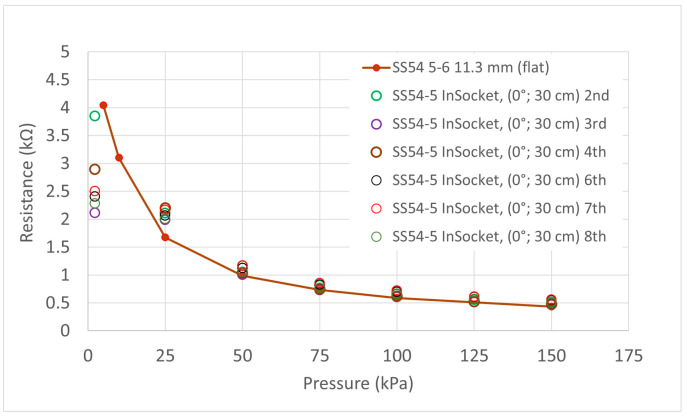
Graph showing in-socket measurement for SS54 Sensel 5 on an area in the socket with a large curvature. The red line curve shows the results obtained for the same sensel on a flat surface and is included as reference.

**Figure 19:**
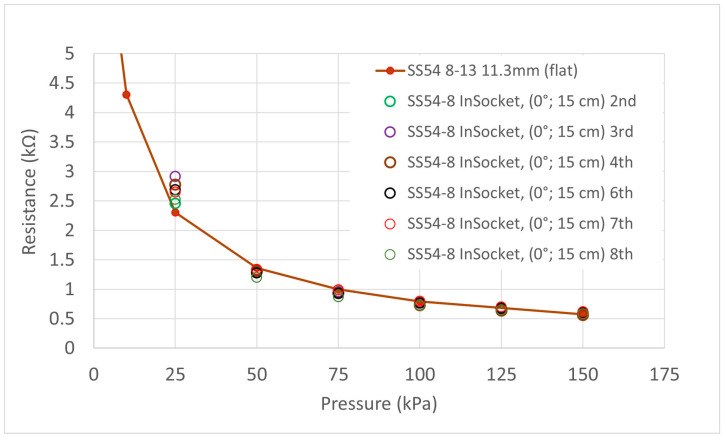
Graph showing in-socket measurement for SS54 Sensel 8 on a relatively flat area in the socket. The red line curve shows the results obtained for the same sensel on a flat surface and is included as a reference.

Eight measurements were done in two sessions. First of all, four measurements were done in a row (1–4), and then eleven days later, another set of four measurements were done in a row (5–8). Typically, 15 min was left between each measurement in each test session, and the liner balloon was removed and replaced between each individual test. The sensor strip was not removed from the socket during the whole period. Two different sensels were studied (S5 and S8). Sensel S5 was placed at an area in the socket with a large curvature, and sensel S8 was placed on a relatively flat area within the socket. Again, the first load in each instance has been treated as a ‘conditioning’ load and disregarded. Figure 18 and Figure 19 show the results for these two tests and a red line resistance versus pressure curve from the testing of the same sensels on the flat has been overlaid as a reference. The output pressures as given by the sensors on the different types of curvatures ‘in-socket’ were generally within 10 kPa of the known pressure generated by the balloon, and therefore, the QTSS™ pressure strip sensors showed a good response to being placed on curvatures.

### 3.4. In-Socket Pilot Testing

**Figure 20:**
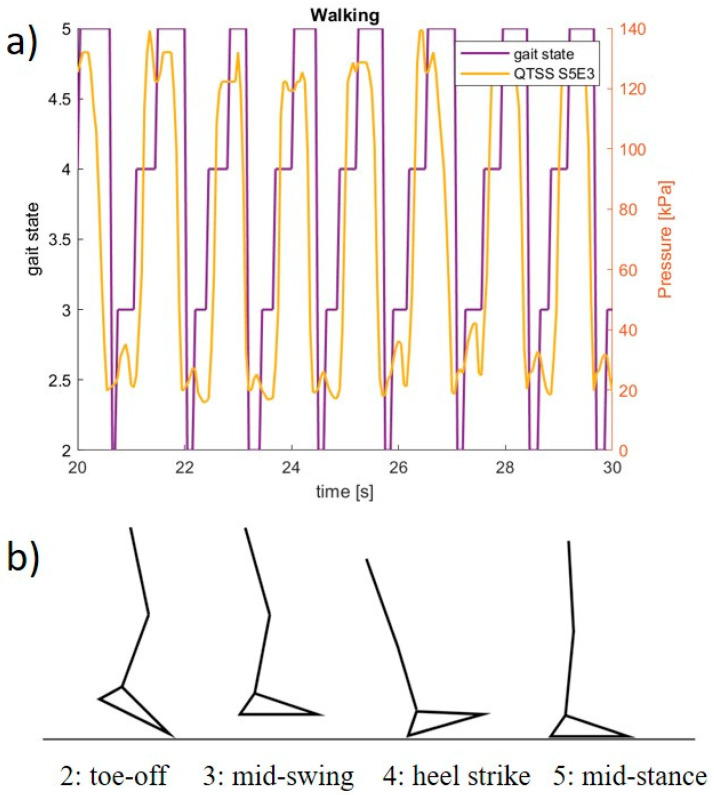
(**a**) An example of a pressure reading output from one sensel on a QTSS™ pressure sensor strip during an initial pilot trial walking gait cycle. (**b**) Gait states: 2 = toe-off, 3 = mid-swing, 4 = heel strike, 5 = mid-stance.

**Figure 21:**
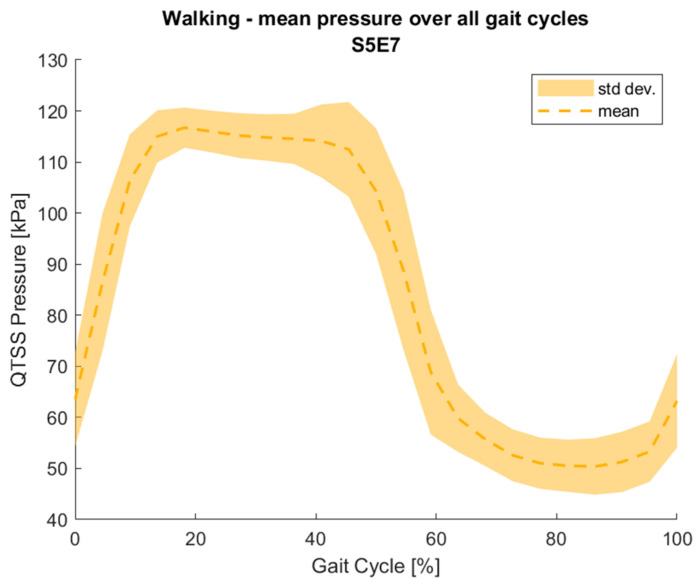
An example of a pressure reading output from one sensel on a QTSS™ pressure sensor strip during an initial pilot trial walking gait cycle.

**Figure 22:**
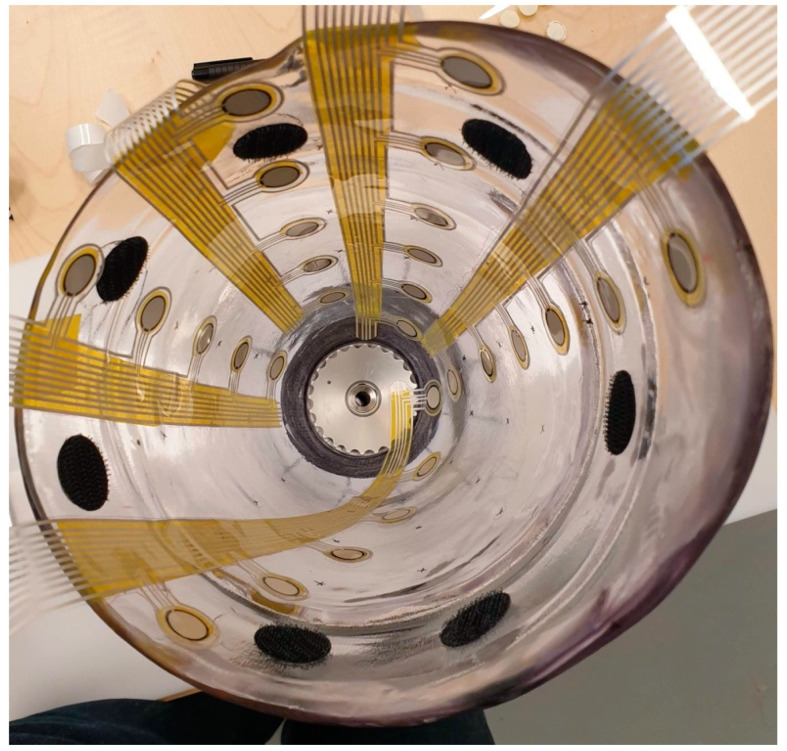
The five QTSS™ pressure sensor strips inside the socket used for the initial pilot trial.

**Figure 23:**
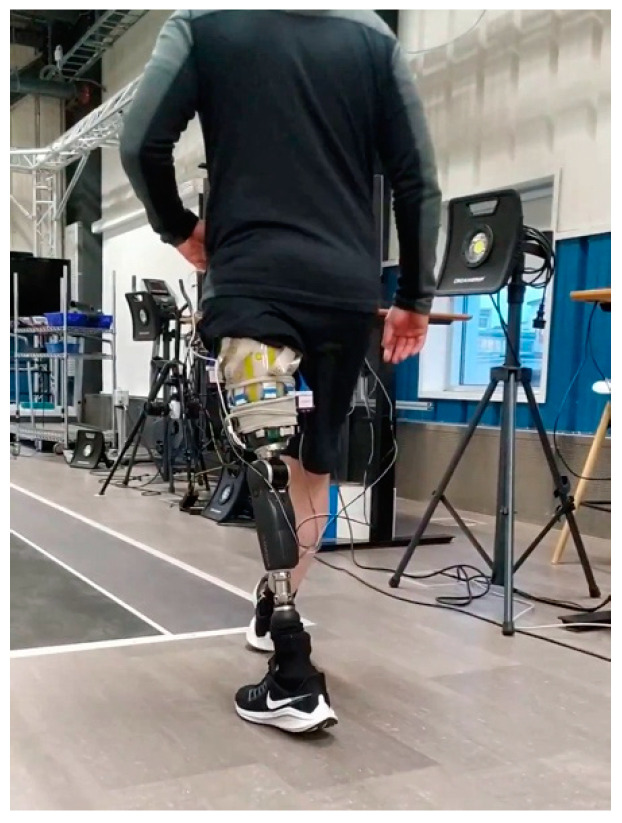
The prototype sensing system on a transfemoral amputee during the initial pilot trial.

The electronics used to create a final prototype sensing system (which currently consists of five QTSS™ pressure sensor strips) primarily comprise a main unit (ESP32 and ESP32 adapter board) and an Analog-to-Digital Converter (ADC) board for each sensor strip. In these electronics, the ADC reads the sensor strip from a voltage divider circuit. The voltage divider circuit used is 3.3 V, and the dividing resistor is 10 kΩ (±1%). The ADC is a 12 bit one, working under a 250 kHz clock. The ADC queries each sensel on a sensor strip 20 times per second, which is the limitation of the sensing system frequency response. The custom-designed software interprets raw data in real time and calculates the pressure–voltage relationship. An initial pilot test was carried out using the final prototype sensing system on a trans-femoral lower limb amputee and the following Figure 20 and Figure 21 show an example of the outputs from a sensel (S5E3 and S5E7) of a QTSS™ pressure sensor strip during a walking gait cycle. An inertial measuring Össur gait monitor was used to determine the gait states, which are described in Figure 20. The configuration of the five QTSS™ pressure sensor strips inside the socket used for this initial pilot trial is shown in Figure 22. Figure 23 shows the prototype sensing system on a transfemoral amputee during this initial pilot trial. The amputee’s socket was an Össur Direct Socket TF [19] and the prosthetic limb consisted of an Össur Reflex rotate [20], an Össur Rheo Knee XC [21], and an Össur ICEROSS transfemoral locking liner was used [22]. VSP Lee et al. [23] presents a similar profile to Figure 21 for a sensel in a similar location in its Figure 6 (labeled R4ANT).

### 3.5. Assembly of Prototype Shear Sensor

**Figure 24:**
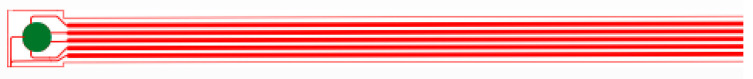
A prototype shear sensor drawing.

**Figure 25:**
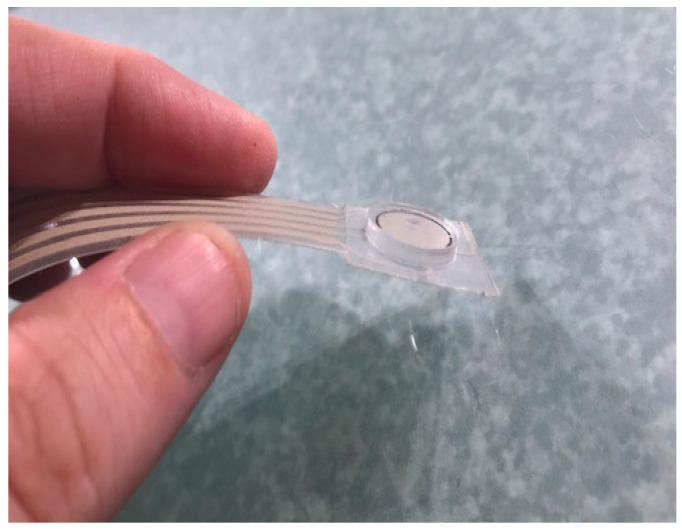
A prototype shear sensor.

The prototype QTSS™ shear sensor is also printed onto PET and is interchangeable in a socket with a QTSS™ pressure sensor strip (see Figure 24 below). Trimmable to any length, it can be placed at key locations in the socket and is to be used to indicate an increasing amount of shear force and the direction of resultant tangential forces. It has four printed silver ink petal electrodes placed below a printed silver ink and QTSS™ ink central electrode. A silicone puck is adhered to the outside of the assembled shear sensor, and the puck creates the contacts when the sensor is in operation (see Figure 25 below). The puck faces the stump once the shear sensor is adhered to the socket wall. Tangential force results in resistance change between the center and peripheral electrodes so that increasing shear forces can be indicated. The four petal electrodes around the edge represent orientations and are read simultaneously to indicate the resultant ‘direction’ of those shear forces.

A 5-way female connector (Amphenol FCI 5-way IDC Connector) has been used to match the 9-way QTSS™ pressure sensor strip connectors so that one shear sensor can be swopped for one pressure sensor strip. The shear sensors are used alongside the pressure sensors to gather data.

Due to the electrode layouts (4 petals), a shear sensor is mounted vertically or at 45 or 90 degrees as those angles are most easily interpreted.

### 3.6. Experimental Setup and the Test Results for the Prototype Shear Sensor

**Figure 26:**
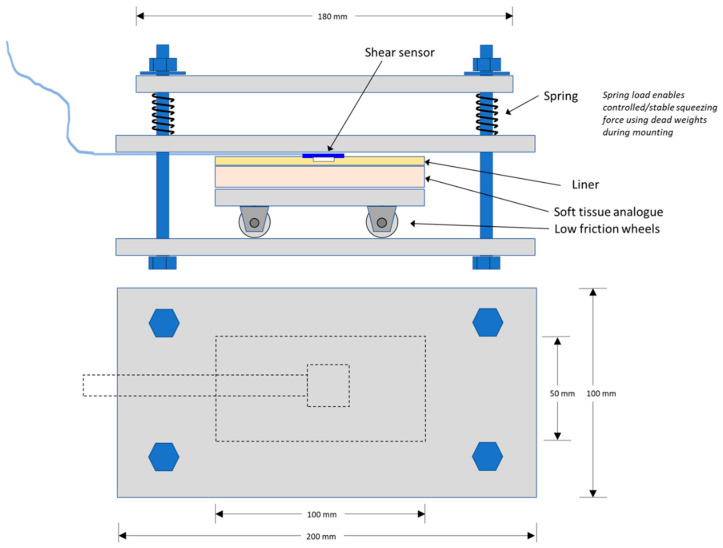
The design and arrangement of the shear sensor test rig.

**Figure 27:**
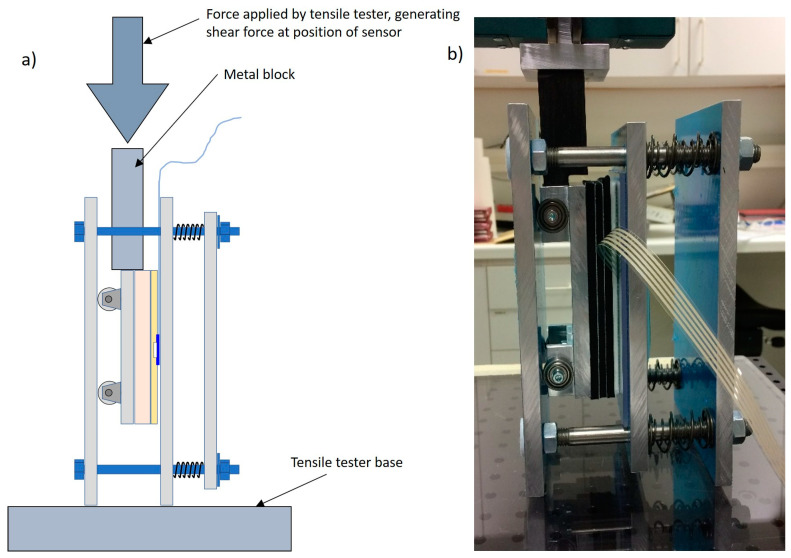
A principle drawing (**a**) and a photo (**b**) showing the shear sensor test rig with the tensile tester during testing.

A shear sensor test rig was developed and built that allowed a QTSS™ shear sensor to be subjected to a shear force when loaded in a tensile tester whilst at the same time being subjected to a controlled normal force. The shear sensor is adhered to the hard top plate and then a large thick pad was placed over the sensor made up of a socket liner material over a block of thick flexible material to replicate the socket liner and the stump skin/muscle. The design and arrangement of the shear sensor test rig and tensile tester is shown in Figure 26 and Figure 27 below.

**Figure 28:**
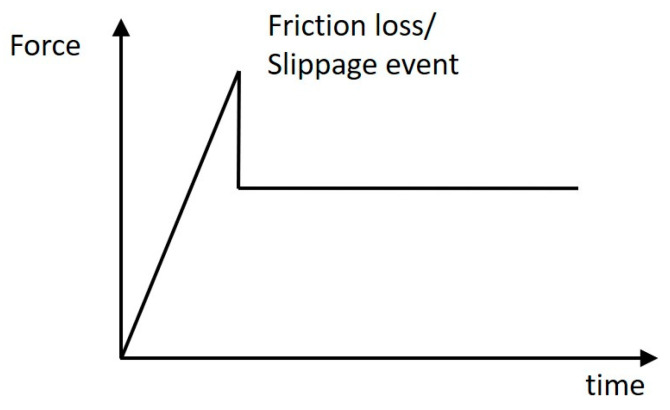
Principle drawing showing a loss of friction/slippage event.

As well as indicating increasing shear forces and the direction of the resultant shear force, a shear sensor can also be used to get an indication of a loss of friction/slippage event within the socket. When a mass (i.e., the stump in the liner) is not moving, it experiences static friction against the shear sensor adhered to the wall of the socket. The friction increases as the applied force increases until a loss of friction/slippage event occurs i.e., on the shear sensor. After this loss of friction/slippage event, the stump experiences kinetic friction, which is less than the maximum static friction. This type of event is depicted in the drawing in Figure 28 below.

**Figure 29:**
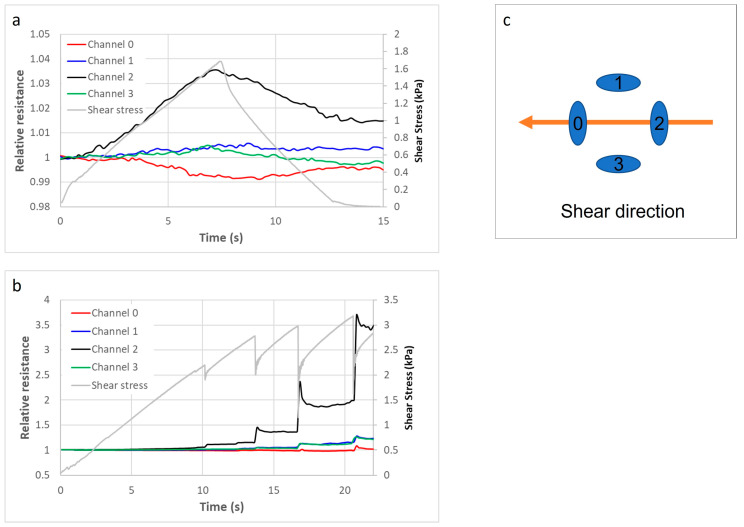
Example results from tests on a QTSS™ shear sensor, using a normal force of 13.6 kPa, without slippage (**a**) and with a series of slippage events (**b**) using a shear force in the direction illustrated in (**c**).

The following graphs in Figure 29 are an example result from a test on a QTSS™ shear sensor using the shear test rig. A normal force of 13.6 kPa has been applied to the sensor, and then, an increasing shear force has also been applied to it from the channel 2 direction toward the channel 0 direction. The relative resistance has been placed on the y axis of the graphs in Figure 29 and gray lines have been overlaid on the graphs to show the application of the shear stress and the slippage events. The direction of the resultant shear force and the increasing shear force is indicated on the top graph in Figure 29 by the black line (channel 2) and the red line (channel 0). The loss of friction/slippage events are indicated on the bottom graph in Figure 29. Further testing and optimization of these sensors will continue during the SocketSense project [12] to ensure that they can withstand physical operating conditions within different sockets and to identify any limitations.

## 4. Conclusions

The QTSS™ sensors presented in this article show excellent potential to be used as thin, low cost, low-weight, real-time prosthetic-fit pressure, shear, and loss of friction sensors in order to address the current healthcare need for improved prosthetic socket fit provision for the masses whilst addressing society’s increasing concerns regarding the environmental impact of electronics and IoT devices.

Simple to manufacture prototype QTSS™ pressure sensors and QTSS™ shear sensors have been developed and assembled using more enviro-friendly and energy-efficient magnetite-based quantum materials. Initial tests have been carried out and have demonstrated that the prototype pressure sensor strip is capable of measuring pressure both on the flat and on curved socket surfaces and that the prototype shear sensor is capable of indicating increasing shear forces, the resultant direction of the shear forces, and loss of friction/slippage events. It is envisaged that these two sensors can be used alongside each other in a prosthetic socket and in the future could be combined into one low-cost simple printed sensing strip. These sensors also have the potential to be used in other situations where there is interfacing between skin and mechanical devices. This work has formed part of the SocketSense project to assist prosthetic comfort and fit [12], and work is continuing on the further testing and optimization of these flexible low-cost prototype sensors presented in this article.
